# Powering Up: a qualitative evaluation of the art and science of meaningful co-production for health equity

**DOI:** 10.1136/bmjpo-2025-003943

**Published:** 2026-07-10

**Authors:** Guddi Singh, Mary Salama, Hannah Zhu

**Affiliations:** 1 Centre of Public Policy Research, School of Education, Communication and Society, King’s College London, London, UK; 2 WHAM (Wellbring and Health Action Movement), London, UK; 3 Birmingham Women's and Children’s NHS Foundation Trust, Birmingham, UK; 4 Evelina London Children’s Hospital Paediatrics, London, UK

**Keywords:** Adolescent Health, Health services research, Qualitative research, Ethics, Child Health

## Abstract

**Background:**

Current quality improvement (QI) efforts rarely engage with the structural conditions that sustain health inequalities. *Powering Up* tested whether creative co-production—using arts, storytelling and citizen science—could move beyond tokenism to meaningfully involve young people (YP) experiencing health inequalities in shaping more equitable paediatric care.

**Methods:**

Conducted in Birmingham and London, the study compared two creative co-production models: *LISTEN UP!* (science-based) and *SHOW UP!* (arts-based). Across both sites, 116 participants (aged 13–18, plus clinicians) engaged in participatory workshops, youth-led investigations and co-produced public outputs. In this process evaluation, we used feminist, community-based and constructivist principles to undertake a reflexive thematic analysis of creative artefacts, observations and participant reflections to explore how co-production was implemented, what supported or constrained it and what was learnt about embedding equity-enhancing participation.

**Results:**

Creative co-production catalysed transformation through three mechanisms—*Education, Empathy and Expression*—which validated lived experience, deepened trust and redefined whose knowledge counts in healthcare. YP reported increased confidence and systemic belonging; clinicians gained insight into social determinants and relational care. Yet entrenched *Intellectual, Institutional and Interpersonal* barriers—limited time, emotional labour and structural misalignment—restricted wider uptake. Sustaining this work required extensive unpaid and affective labour, resulting in moral distress and burnout among facilitators. This revealed a paradox: co-production as a *labour of love* operating within systems whose structures make that labour unsustainable.

**Conclusions:**

Meaningful co-production is not a technical fix, but a moral and political practice grounded in *Respect, Relationships and Reciprocity*. While creative methods can catalyse relational healing and systemic insight, their continuation demands policy-level recognition, funding reform and protection of the human infrastructure that makes participation possible. Without structural investment, co-production risks remaining an ethical aspiration performed in unsustainable conditions—an institutional hypocrisy that must be addressed for health systems to deliver equity.

**WHAT IS ALREADY KNOWN ON THIS TOPIC**

- Co-production is widely promoted in healthcare but often implemented superficially, with limited attention to the labour, time and relational infrastructure it requires.
- Its potential to advance equity, particularly in paediatrics and underserved communities, remains under-realised.

**WHAT THIS STUDY ADDS**

- Creative, power-sharing approaches can foster trust, mutual learning and systemic insight between young people and clinicians.
- However, the work’s sustainability depends on extensive unpaid and emotional labour—revealing a structural hypocrisy in systems that celebrate co-production while failing to resource it.

**HOW THIS STUDY MIGHT AFFECT RESEARCH, PRACTICE OR POLICY**

- Highlights the need for national and organisational policies that fund and protect relational, equity-focused models of co-production.
- Calls for performance metrics and QI frameworks that value relational and affective labour as essential system-shaping work.
- Positions co-production as moral infrastructure, requiring investment if it is to move from rhetoric to reality.

## Introduction

 Health inequalities persist despite decades of research and reform. While health systems research has documented the extent and impact of disparities,^1^ many clinical quality improvement (QI) initiatives still give limited attention to the structural, relational and cultural dynamics that sustain them.^2^ Co-production—defined as the active involvement of patients and communities in designing and delivering healthcare—has been proposed as one response.^3 4^ Yet, in practice, co-production often falls short of its transformative promise.

Marginalised youth are those who experience social, economic, cultural or structural disadvantage, including poverty, racism, discrimination, exclusion from services or reduced power within institutions.^5 6^ Their perspectives are often missing from healthcare research and improvement work, despite the fact that young people from the most deprived communities experience complex, intersecting health needs and disproportionately poor outcomes.^5^ Paediatrics therefore provides a revealing lens on how health inequalities are created, sustained and encountered in everyday care.

Conventional QI efforts often sidestep these complexities, focusing on operational efficiency or clinical effectiveness while marginalising questions of inclusion, justice and power.^7^ Co-production may reproduce existing hierarchies when it privileges already-engaged participants, reduces involvement to consultation or fails to challenge professional authority.^8^,^10^ Existing participatory work is therefore often limited by exclusionary participation, insufficient relational continuity and the marginalisation of experiential knowledge.^11^,^14^ What is needed is not simply more co-production, but better co-production: rooted in equity, trust and shared ownership.

Creative participatory methods offer one route towards this deeper form of engagement. Storytelling, arts-based practice and performance can surface lived experience, create less formal spaces for dialogue and engage young people who may feel alienated by conventional consultation processes.^15^,^19^ However, these methods remain relatively uncommon in clinical improvement work, where experiential and creative knowledge may be marginalised by dominant evidence hierarchies.^13 14^ There is limited empirical work examining how creative methods can be embedded in co-production for health equity, particularly with marginalised young people.

The *Powering Up* pilot addressed this gap by bringing together young people, clinicians, researchers and community partners to explore creative co-production in paediatric health. The project compared two contrasting approaches—LISTEN UP!, a science-based citizen inquiry model, and SHOW UP!, an arts-based model using storytelling, performance and visual methods. This paper reports the process evaluation, asking what resources were required, what benefits and barriers emerged and how creative practices shaped power, trust and participation.

Drawing on feminist and relational traditions, we treat co-production not as a neutral technique but as an ethical and political practice of power-sharing. The aim of this paper is to offer a practice-based account of what creative co-production can make possible, what constrains it and what systems must recognise if equity-focused participation is to move beyond rhetoric.

## Methods

### Theoretical orientation

Powering Up was grounded in a participatory, relational and critically informed approach to health systems improvement.^20^,^22^ We understood health inequalities as structurally produced and unevenly experienced, and co-production as an ethical practice of power-sharing rather than a neutral engagement technique.^10 14 23^ This orientation drew on feminist theory, community-based participatory research and critical pedagogy,^20^,^22^ and shaped the study design, creative methods, reflexive analysis and attention to relational ethics. In practical terms, it led us to treat lived experience, emotion, creative expression and silence as analytically meaningful forms of data, alongside interview, survey and observational material.^15 16 23 24^

### Study design

Powering Up was a QI initiative and process evaluation exploring how creative co-production might support more equitable participation in paediatric healthcare. Rather than testing a predefined intervention under controlled conditions, it examined two co-production approaches operating within real-world service and community settings. The design was therefore opportunistic and practice-embedded, recognising that meaningful participation must work within existing system constraints.

The evaluation was guided by SQUIRE 2.0^25^ and informed by community-based participatory research, feminist theory and critical pedagogy.^23^ These approaches emphasise lived experience, shared decision-making and attention to power in knowledge production.

**Figure 1:**
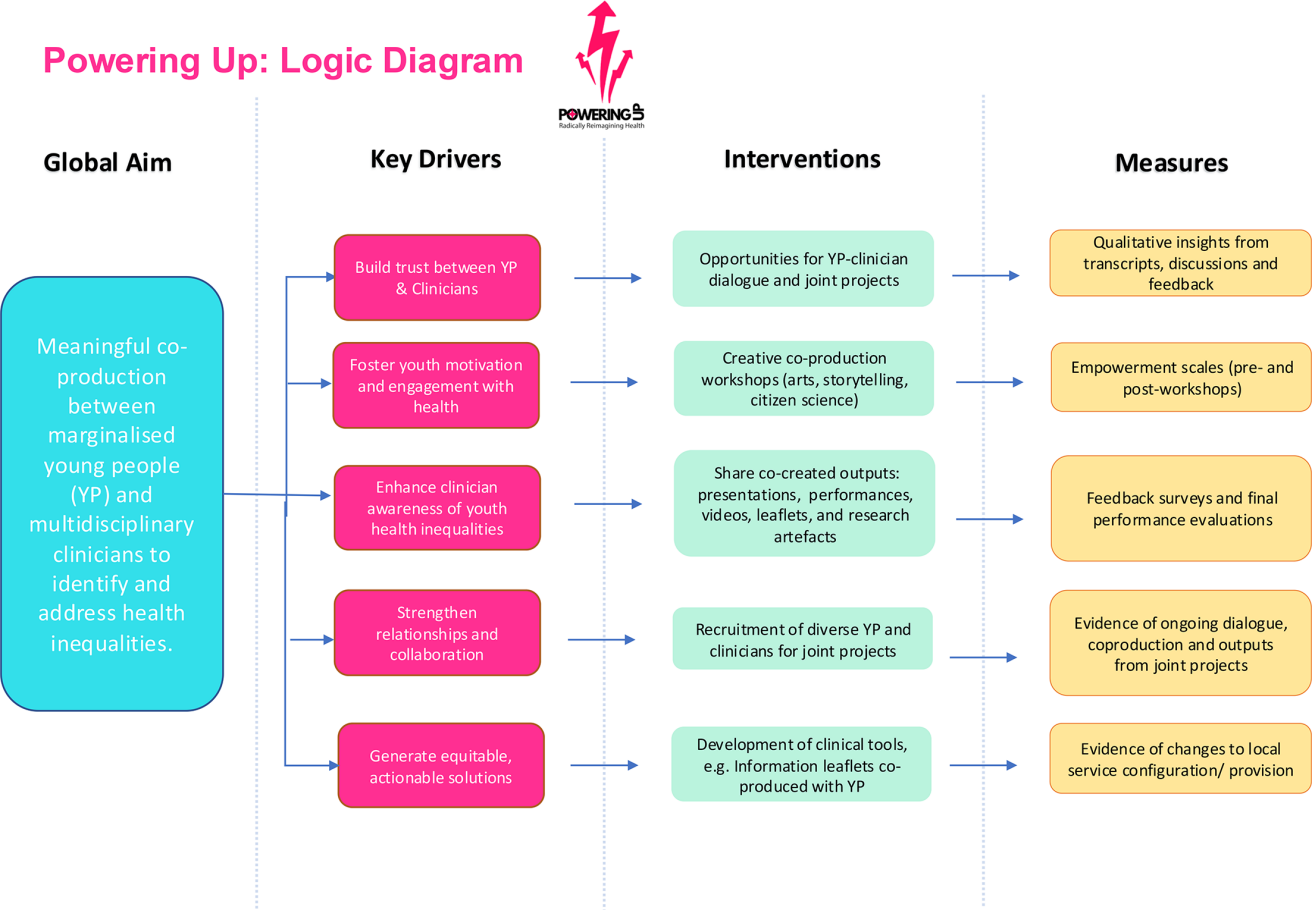
Logic diagram of Powering Up’s key driver, interventions and measures.

Two intentionally contrasting models were implemented (figure 1). LISTEN UP! used science-based citizen inquiry to engage larger numbers of young people through shorter, structured encounters. SHOW UP! used arts-based methods, including theatre, storytelling, music and visual work, to engage smaller groups in more sustained relational work. Both arms shared a commitment to equity, lived experience and systemic critique, enabling comparison of how method, context and capacity shaped the possibilities of co-production.

### Settings and participants

The project was implemented in Birmingham and London—two ethnically diverse and socioeconomically unequal UK cities that provided relevant contexts for examining health inequities and system responsiveness. Participants included 116 young people aged 13–18 and 11 clinicians. Schools were used as inclusion sites to reach young people from highly deprived communities, including those less likely to engage through traditional healthcare routes. Clinicians were recruited nationally from diverse specialties and professional levels.

Across both strands, participants were positioned as co-creators rather than research subjects. Young people contributed to the project’s visual identity, creative outputs and shared reflections with clinicians and system stakeholders. The pilot intentionally compared two contrasting approaches: LISTEN UP! as a ‘wide-but-shallow’ model, involving many young people through shorter encounters, and SHOW UP! as a ‘deep-but-narrow’ model, involving fewer young people and clinicians through more intensive engagement. This design allowed us to explore how different forms of creative co-production operated within real-world constraints of time, resource and institutional capacity.

Participant characteristics and project activities are summarised in table 1.

**Table 1:** Comparison and methodological overview of the two Powering Up models

Feature	LISTEN UP! Science—Birmingham	SHOW UP! Arts—London
Purpose	Increase young people’s awareness of health inequalities and develop citizen inquiry skills	Envision responses to health inequalities through creative co-production
Methodological approach	Science-focused citizen inquiry using classroom discussion, community data collection and youth-led presentation	Arts-based co-production using theatre, storytelling, music, visual arts and performance
Participants	100+ school students aged 13–18 years across three schools	14 students aged 13–18 years and 11 clinicians
Delivery partners	Canal Project, Birmingham City Council and Birmingham Children’s Hospital, led locally by the Community Engagement Lead	WHAM and Collage Arts at Dunraven School, with final performance at Science Gallery London
Structure and contact	Three 60–90 min school-based touchpoints over 2 months, plus a final youth-led stakeholder event	Five consecutive full-day workshops, with individual and joint sessions involving young people and clinicians
Core activities	Classroom inquiry, community research, theme exploration, ergonomic modelling and presentation to NHS/public health leaders	Co-facilitated creative workshops using dance, poetry, drama, storytelling, drawing and performance
Outputs	Youth-led community findings presented to NHS and public health leaders	Multimodal creative outputs including film, live performance, music, poetry and website material
Relational intensity	Wider reach but shorter contact; relationships largely limited to project sessions	Smaller reach but deeper engagement; relationships extended beyond workshops with ongoing digital contact
Adaptive flexibility	Built around structured inquiry while allowing young people to collect and present community perspectives	Adapted responsively to participants’ ideas, emotions and creative preferences

NHS, National Health Service; WHAM, Wellbeing & Health Action Movement.

### Data collection

Guided by a social constructivist and participatory epistemology, data collection was designed to capture not only *what* participants said or produced, but *how* meaning, power and emotion were negotiated through creative co-production. We used a multimodal qualitative design integrating creative artefacts, participant observation, interviews and reflective conversations, fieldnotes, feedback surveys and video diaries. Table 2 summarises the data corpus used in the process evaluation, including the approximate volume and purpose of each data source. These data were generated throughout delivery rather than at a single time point, enabling us to examine how relationships, perceptions and experiences evolved across the two co-production models.

**Table 2:** Data collection overview

Method	Participants/context	Approximate volume	Purpose
Interviews/reflective conversations	Young people and clinicians	24	Process reflection
Participant observation	Workshops and public events	16 sessions	Contextual understanding
Creative outputs	Young people	46 artefacts	Embodied insights
Reflective fieldnotes	Research team	33 records	Interpretive depth
Feedback surveys	Participants	37 responses	Immediate and longitudinal responses
Video diaries	Youth participants	11 diaries	Sustained longitudinal voice

Data collection was iterative and responsive to participants’ preferences and the unfolding relational context at each site.^26 27^ Different sources served complementary purposes: creative outputs surfaced embodied and affective knowledge; observations captured interpersonal and contextual dynamics; interviews, surveys and video diaries elicited evolving reflections; and fieldnotes and analytic memos documented researcher interpretation and positionality. Team debriefs, participant feedback loops and analytic journaling supported reflexivity, triangulation across data sources and comparison between LISTEN UP! and SHOW UP!, while remaining attentive to each model’s distinctive affordances and constraints.

### Data analysis

Data were analysed using reflexive thematic analysis, which is well suited to feminist, constructivist and participatory inquiry because it treats interpretation as an active process of meaning-making rather than neutral extraction of themes.^26 27^ The analytic team included clinicians, social scientists, artists, medical students and youth researchers; these varied perspectives were treated as reflexive resources.

Analysis involved repeated reading, viewing and discussion across the full dataset, followed by manual coding by at least two team members, comparison across data sources and collaborative refinement of candidate themes. Coding was primarily inductive, grounded in participant narratives and creative outputs, while also informed by our theoretical commitments to feminist ethics, relational care and equity. Creative, observational and textual data were compared with examine convergence, divergence and site-specific patterns across LISTEN UP! and SHOW UP!

Our findings—organised as the ‘3 Es’, ‘3 Is’ and ‘3 Rs’—were not pre-existing coding categories, nor discrete participant-generated themes. They were developed during later-stage reflexive synthesis as interpretive heuristics to organise recurrent patterns across multiple data sources and sites. In keeping with reflexive thematic analysis, these frameworks represent analytic constructions developed through iterative engagement between data, theory and researcher interpretation.^26 27^

Trustworthiness was supported using Lincoln and Guba’s criteria for naturalistic inquiry.^28^ Credibility was strengthened through prolonged engagement, triangulation and participant feedback; dependability through analytic memos and theme maps; confirmability through reflexive journaling and team discussion; and transferability through rich contextual description. Divergent perspectives were documented rather than resolved.

### Ethical and reflexive considerations

This project was undertaken as a QI initiative rather than a conventional research study. Its primary purpose was to improve local practice and explore the implementation of co-production within real-world settings rather than generate generalisable causal knowledge. Governance and ethical oversight were therefore undertaken through local QI processes, in accordance with UK Health Research Authority guidance.^29^

Our ethical approach was also informed by relational ethics and care ethics traditions.^21 30^ Consent was treated as layered and ongoing, with opportunities for participants to opt in, opt out or renegotiate involvement. Particular care was taken around confidentiality, representation and ownership of creative outputs, especially in digital and public-facing dissemination. Quotations and creative materials used for dissemination were reviewed with participants to minimise identifiability and ensure agreement.

Reflexivity was woven through team debriefs, analytic memos and participant feedback loops. We remained attentive to risks of tokenism, emotional burden and institutional limits to power-sharing. The research team was multigenerational and professionally diverse, with leadership primarily from medical professionals; no participating young person had a clinical relationship with any clinician involved in the project.

### Patient and public involvement

Young people were involved from project inception, shaping priorities, co-designing workshop activities and outputs, informing process indicators, supporting inclusive recruitment and co-producing dissemination through performances and stakeholder presentations. Findings will be shared back through school-based and community-based events and online resources.

## Findings

This pilot compared two complementary co-production models—LISTEN UP! Science and SHOW UP! Arts—that engaged marginalised young people in shaping more equitable health and care systems. Although the models differed in structure and intensity, both fostered trust, mutual learning and opportunities for young people to influence professional understanding. LISTEN UP! prioritised breadth through shorter encounters with larger numbers of young people; SHOW UP! prioritised depth through more sustained relational engagement.

### Material matters: how structure shapes possibility

The two models differed not only in creative method but also in their demands on participants, clinicians and organisations (table 1).

LISTEN UP!, grounded in citizen science, followed a structured process of discussion, fieldwork and youth-led presentation. Clinicians frequently described the format as easier to integrate into existing professional commitments:

I believe in this. But as a consultant? My diary’s a war zone. Even asking to do this feels radical. (Consultant clinician)

Young people nevertheless reported increased confidence and trust in healthcare professionals:

That doctor didn’t seem scary by the end… I was asking them questions, and I felt like they really saw me. (Young person, 15 years)

SHOW UP! used storytelling, performance and creative expression to support deeper relational engagement. Although involving fewer participants, it generated more sustained interaction between young people and clinicians. Participants frequently described shifts in understanding and connection:

Spending a day with kids as people, not patients? Game-changing. Now I understand their clinic behaviour so much better. (Junior doctor)

### The benefits of creative co-production: the 3 Es

Across both sites, participants consistently described experiences of learning, perspective-taking, validation and voice. Through reflexive analysis, these recurrent patterns were synthesised into three interrelated mechanisms of change: Education, Empathy and Expression (table 3).

**Table 3:** The 3 Es of empowerment

E of empowerment	What it did	Illustrative quotes
Education	Reframed knowledge; young people became teachers, clinicians became learners	“It’s made me want to learn more about my community and why we are unhealthy.” (Young person, Birmingham) “I believe I could work in the NHS now. I wouldn’t have seen myself as a scientist before, but I did it!” (Young person, Birmingham) “I learned more today than in years of training. Real stories are so much more effective than PowerPoints.” (Junior doctor)
Empathy	Humanised both groups; built trust and mutual recognition	“I thought doctors would be boring. Turns out they’re just people—funny, and with feelings, like us. Some of them are really nice.” (Young person, 16 years) “It’s the first time I understood what it means to be a poor, Black, male in London today. And it’s heartbreaking.” (Junior doctor) “It’s amazing being on our own turf. I feel like we’re in charge and the doctors are not. I’m not sure I would have done any of this if we’d had to come to the hospital.” (Young person, 18 years)
Expression	Enabled voice beyond words; creative methods offered agency, especially for those marginalised by language or literacy norms	“Thank you for letting us express ourselves differently. I’m not good with words, but when I dance, I feel alive and able to speak my truth.” (Young person, 16 years)

Together, these mechanisms helped participants move beyond conventional consultation, creating opportunities for young people to contribute knowledge, build confidence and engage more directly with clinicians and systems.

### Barriers to embedding equity-focused co-production: the 3 Is

Alongside these enabling mechanisms, participants and facilitators described recurring barriers relating to knowledge systems, organisational structures and relationships. These were synthesised into three interconnected domains of challenge: Intellectual, Institutional and Interpersonal barriers (table 4).

**Table 4:** The 3 Is: barriers to equity-focused co-production

Barrier	Core challenge	Illustrative quotes
Intellectual	Professional discomfort with ambiguity, emotional knowledge and non-hierarchical methods	“Honestly? No clear rules stressed me out. I’m used to being told what to do. Leading felt scary—what if I messed up?” (Clinician, medical student) “Arts and health inequalities? I was sceptical. Now? I see how it bridges gaps textbooks never could.” (Consultant clinician)
Institutional	Structural misalignment: time, roles, metrics and cultural norms inhibited sustainability	“Love this idea, but implement it? With our workloads? I’m barely surviving as-is.” (Clinician) “Colleagues wanted to come. Their bosses said no. That tells you everything.” (Junior doctor)
Interpersonal	Emotional risk and relational vulnerability; past marginalisation and burnout shaped trust	“I loved this, but showing vulnerability to patients? Still feels unnatural. I’m not there yet.” (Paediatrics trainee) “This project felt special—I could really speak up here. But with my actual GP? It’s never like this.” (Young person, 16 years)

These barriers were evident across both sites, although their expression varied according to organisational context, available resources and existing relationships.

### Impacts: change across people, systems and possibility

Creative co-production generated impacts across young people, clinicians and systems. Rather than functioning only as ‘engagement’, participants described it as doing three kinds of work: *therapy*, by enabling expression, connection and relational repair; *prevention*, by building trust and confidence in healthcare relationships; and *intelligence*, by surfacing lived knowledge and system blind spots usually hidden from formal improvement processes (table 5).

**Table 5:** Impacts and functions of creative co-production

Level of impact	Observed impact	Function of co-production	Illustrative quote
Young people	Increased confidence, connection, civic agency and willingness to speak into systems	**Therapy:** expression and connection supported relational repair and belonging **Prevention:** improved trust made future help-seeking feel safer	“Speaking honestly here felt powerful. But my GP’s office? Doubt they’d get it. This room was special.”
	Reduced fear of clinicians and greater confidence navigating healthcare spaces	**Prevention:** embodied encounters with clinicians reduced fear and created conditions for earlier, more confident engagement with care	“Going to the doctor is not scary anymore.”
Clinicians	Emotional renewal, deeper insight into young people’s lives and changed understanding of social determinants	**Intelligence:** lived experience surfaced knowledge not usually visible in clinical encounters. **Therapy:** relational work also restored professional purpose	“I feel I can do a better job for my patients now—this kind of understanding should be part of our training.”
	Frustration and moral discomfort when returning to unsupported clinical systems	**Intelligence:** exposed the gap between what clinicians value and what systems make possible	“It’ll be sad to go back to real life—we can’t do this in our day jobs.”
Systems	Collaboration across sectors, cultural shift and bottom-up insight into service barriers	**Intelligence:** generated system knowledge from lived experience and revealed where services failed to connect	“This is the first time I’ve heard what it’s actually like to access services I work in. It changes everything.”
Cross-cutting	Participants described the work as emotionally reparative, trust-building and insight-generating	**Therapy, prevention and intelligence** operated across individuals, relationships and systems rather than belonging to any one group	“To many, it feels like home.”

These functions were not separate outcomes but overlapping effects of relational co-production, helping explain why participants experienced the work as both personally meaningful and systemically revealing.

### The values that matter: the 3 Rs

While creative tools mattered, participants consistently emphasised that meaningful co-production depended on three relational conditions: *Respect, Relationships and Reciprocity* (table 6). These were not abstract values but practical ways of working. Across both sites, young people repeatedly described trustworthy professionals as those who were ‘real’: emotionally honest, realistic about limits and reliable in their follow-through. These qualities cut across the 3 Rs, helping explain why some forms of participation felt safe and mutual, while others risked feeling extractive.

**Table 6:** The 3 Rs: relational conditions for meaningful co-production

Relational condition	What it required in practice	What ‘being real’ meant to young people	Illustrative quote
Respect	Recognising lived experience as expertise; avoiding tokenism; being transparent about purpose and power	Being emotionally honest and human, rather than hiding behind professional distance	“Treat us like partners, not inspiration quotes.”
Relationships	Building trust over time through repeated contact, humour, vulnerability and follow-through	Being reliable: returning when promised, staying present, and showing that the relationship was not a one-off performance	“They actually came back the next week, like they said. That’s rare.”
Reciprocity	Making exchange mutual: young people contributed knowledge while clinicians also listened, reflected and risked vulnerability	Being realistic: honest about limits, constraints and what could or could not change	“Doctors had to perform too—not just us. Felt good seeing them on our level.”

These relational conditions were fragile and had to be built through time, honesty and shared vulnerability; without them, co-production risked becoming another form of symbolic participation.

## Discussion

Powering Up offers a practice-based comparison of how creative co-production with marginalised young people can contribute to more equitable health systems. Across two contrasting models, the findings show that creative co-production can generate trust, learning and system insight, but only when supported by the relational and material infrastructure required to make participation meaningful rather than symbolic.

### Creative co-production as epistemic repair

Our analysis identified three interlocking mechanisms—Education, Empathy and Expression (the 3 Es)—through which creative co-production moved beyond tokenistic engagement to reshape how knowledge is shared and valued. Rather than inviting participation into pre-existing professional frames, these mechanisms reconfigured who was seen as credible and what counted as expertise.

This matters because conventional QI can inadvertently reproduce inequity when it privileges efficiency, standardisation and professional expertise while marginalising lived experience.^31^ By legitimising experiential, emotional and creative knowledge alongside clinical expertise, Powering Up responded to what Fricker describes as epistemic injustice: the exclusion or downgrading of knowledge because of who offers it.^32^ In doing so, it echoes Groot *et al*’s call for more ethical forms of co-production that actively rebalance epistemic authority rather than simply collecting patient perspectives.^33^

The value of creative discomfort was one of the study’s most striking insights. Co-production ‘worked’ not because it smoothed over differences, but because it created safer conditions in which difficult truths could be surfaced and examined. Young people challenged professional assumptions and institutional language, while clinicians encountered forms of knowledge and experience that sat beyond conventional biomedical training. This productive friction aligns with Robert *et al*’s account of co-production as a space where transformation emerges through discomfort rather than consensus,^10^ and with Flinders *et al*’s argument that challenge and disruption are often prerequisites for meaningful change.^14^

Across both sites, learning flowed in multiple directions. Young people were positioned not as patients, consultees or data sources, but as knowledge-holders whose experiences could reshape professional understanding. Clinicians, in turn, stepped into learner roles. This reciprocity mirrors Cribb *et al*’s vision of co-creation as a space where expertise is shared, relational and dynamic, rather than fixed by professional status.^34^

### The hidden cost of meaningful co-production

Alongside these enabling mechanisms, the 3 Is—Intellectual, Institutional and Interpersonal barriers—help explain why equity-focused co-production remains difficult to embed despite widespread rhetorical support.

Intellectual barriers reflected uncertainty about whether experiential, emotional and creative knowledge would be taken seriously within systems privileging biomedical evidence.^35^ Institutional barriers were most significant: workforce pressures, lack of protected time, limited funding and fragmented engagement infrastructure meant that relationships were difficult to sustain beyond the funded pilot.^36 37^ Interpersonal barriers reflected the emotional labour required of professionals and young people as they navigated vulnerability, mistrust and uncertainty.^14^

Importantly, these barriers did not operate independently. Rather, they interacted to create conditions in which good intentions frequently struggled to translate into sustained practice. Clinicians working within overstretched services often lacked the organisational support needed to act upon the insights generated through co-production. Likewise, young people who experienced meaningful participation frequently encountered systems unable to maintain the relationships they had helped create. Without protected time, relational capacity and sustainable funding, co-production risks becoming a labour of love performed within systems that make such labour unsustainable.

### From participation to systemic belonging

Beyond individual learning, creative co-production appeared to function as a rehearsal for systemic belonging. Through education, empathy and expression, young people experienced themselves as contributors to health systems rather than passive recipients of care. Holding activities in schools and community settings, rather than clinical spaces, helped decentre institutional power and made participation feel safer, more accessible and more relevant to everyday life.

These shifts align with calls for medical professionalism to become more attentive to social determinants, structural injustice and community partnership.^38^ They also resonate with MacGregor *et al*’s account of collective capacity building: developing shared resources for transformation rather than locating ‘empowerment’ solely within individuals.^39^

The findings also suggest several reasons why these approaches may have avoided some of the limitations commonly associated with co-production. First, activities were situated within contexts familiar to young people, increasing accessibility and reducing institutional barriers to participation. Second, creative methods disrupted traditional hierarchies by creating spaces in which clinicians and young people could meet as people rather than as professionals and patients. Third, storytelling, performance and citizen inquiry enabled forms of epistemic reconfiguration, positioning lived experience as a legitimate source of knowledge rather than a supplement to professional expertise. Finally, both models relied on iterative and relational engagement rather than one-off consultation events, allowing trust and reciprocity to develop over time.

These insights help explain why Respect, Relationships and Reciprocity emerged as such important findings. The 3 Rs were not abstract values but practical conditions through which meaningful participation became possible. Participants’ repeated emphasis on professionals being honest, realistic and reliable suggests that trustworthiness was not assumed through professional status; rather, it had to be enacted through consistency, vulnerability and follow-through.

### Beyond co-production: towards transformative praxis

Our findings suggest that meaningful co-production is grounded in more than participation alone. It requires a broader rethinking of knowledge, power, time and improvement itself. As Cribb argues, advancing equity requires expansive thinking about institutions, expertise and professional practice.^34^

Building on Beckett *et al*,^40^ the study suggests four practical shifts for equity-focused improvement work. First, expertise must be broadened beyond clinical knowledge to include lived and creative forms of knowing. Second, power must be actively shared rather than simply managed. Third, time must be understood not only as a resource but as a relational condition necessary for trust-building. Finally, success must be evaluated through indicators such as trust, agency and system learning alongside conventional performance metrics. These shifts do not replace traditional QI goals but expand what counts as improvement.

When practiced as a philosophy of system redesign rather than a discrete engagement tool, co-production can catalyse what might be described as collective reflexivity: a shared capacity to see, name and challenge power.^23^ Participants frequently described this as ‘speaking into systems’—not simply being heard but helping reshape what health systems hear and value.

### Transferability, limitations and implications

This pilot illuminated how creative co-production can transform relationships, but its relatively small scale leaves open important questions about sustainability and transferability. As Greenhalgh and colleagues have observed, the NHS often excels at innovation but struggles with permanence.^9^ Future work should therefore examine not only whether young people sustain the confidence, agency and civic engagement fostered through participation, but also whether organisations develop the capacity to maintain the relationships on which meaningful co-production depends.

While the findings offer rich insight into the processes and conditions that support creative co-production, they are not intended as a universally generalisable model. The comparison between LISTEN UP! and SHOW UP! is instructive in this regard. LISTEN UP! appeared more feasible for reaching larger numbers of young people through structured, shorter encounters, whereas SHOW UP! generated deeper relational and affective learning through more intensive engagement. Neither model should be understood as inherently superior. Rather, each revealed different trade-offs between breadth, depth, feasibility and sustainability. Replication would therefore require adaptation rather than simple reproduction, particularly in resource-constrained settings where protected time, skilled facilitation and community trust may be harder to secure.

Future work must consider not only what co-production achieves, but what it costs—and who pays. The success of this pilot relied on significant unpaid labour and relational care far beyond what the small grant formally covered. Even with a highly motivated interdisciplinary team, meaningful co-production demanded extraordinary investments of time, trust and emotional energy. Clinicians participated around existing workloads and workforce pressures; researchers and facilitators undertook substantial relational labour to build, sustain and honour relationships with young people; and the work of maintaining trust frequently extended well beyond funded project timelines.

Perhaps the most striking finding was the extent to which meaningful co-production depended on unpaid, affective and often invisible labour. Participants and facilitators described substantial investments of time, emotional energy and relational work that were rarely recognised within formal organisational structures. The result was a persistent gap between institutional aspiration and institutional support. We describe this as a form of ‘structural hypocrisy’—not to imply malicious intent, but to capture the contradiction between health systems’ stated commitments to equity and participation and the absence of the infrastructure required to sustain them.

This interpretation resonates with several strands of scholarship. Within healthcare improvement, it reflects the familiar gap between work-as-imagined and work-as-done,^11 41^ whereby organisational ambitions frequently exceed the resources available to deliver them in practice. It also echoes Ahmed’s concept of institutional non-performativity, in which commitments to inclusion and participation may be publicly affirmed without creating the organisational conditions necessary for their enactment.^42^ Finally, it reflects a longstanding tension identified within care ethics: institutions depend on caring and relational labour while simultaneously rendering much of that work invisible.^30^

This disconnect points to a troubling gap between aspiration and infrastructure. Clinicians are increasingly encouraged to champion equity, partnership and participation, yet the protected time, funding, workforce capacity and cultural support required to do so are rarely provided. Without structural investment in the people and processes that make co-production possible, its promise risks remaining largely rhetorical.

Embedding co-production as routine, equitable healthcare will require more than enthusiasm. Funding, workforce planning, governance and evaluation frameworks must recognise relationship-building as system-shaping work. National policy, professional education and organisational leadership must treat equity-focused co-production not as an optional add-on, but as a core component of high-quality care.

## Conclusion

### Towards a more just practice of co-production

This study is neither a toolkit nor a one-size-fits-all model. It offers a practice-based account of what becomes possible when creative co-production is used to engage marginalised young people not as subjects of improvement, but as co-creators of knowledge, relationships and system insight.

Across LISTEN UP! Science and SHOW UP! Arts, creative co-production functioned as relational repair, systems intelligence and public health prevention. These benefits required Respect, Relationships and Reciprocity, and were constrained by intellectual, institutional and interpersonal barriers.

The central lesson is that meaningful co-production cannot rest on goodwill alone. It requires medical education that cultivates epistemic humility, funding models that value relational labour, and evaluation frameworks that centre those most affected by inequity. Otherwise, co-production risks becoming what Brady calls ‘equity theatre’: participation celebrated in principle but unsupported in practice.^6^

Yet the pilot also offers grounds for hope. By creating spaces where young people and clinicians could learn, feel, speak and imagine differently together, *Powering Up* rehearsed more just possibilities for paediatric care. As one young participant put it: “Stop calling us ‘hard-to-reach’ when you’re holding the door shut.” This is the challenge co-production must answer: not how to extract more voice from marginalised communities, but how to change the systems that make listening difficult. Co-production done with intention, care and courage should not be treated as an optional add-on, but as infrastructure for equity, trust and transformation.

## Data Availability

Data are available upon reasonable request.
