# Supplementary material for: Powering Up: a qualitative evaluation of the art and science of meaningful co-production for health equity
Source: BMJ Paediatr Open. 2026 Jul 10;10(1):e003943. doi: 10.1136/bmjpo-2025-003943 (PMC13358257; doi:10.1136/bmjpo-2025-003943)
Supplement: Supplementary file 1 [file bmjpo-2025-003943.reviewer_comments.pdf]

PEER REVIEW HISTORY

BMJ Paediatrics Open publishes all reviews undertaken for accepted manuscripts. Reviewers are asked to complete a checklist review form and are provided with free text boxes to elaborate on their assessment. These free text comments are reproduced below.

ARTICLE DETAILS

**TITLE (PROVISIONAL)**	POWERING UP: A qualitative evaluation of the art and science of meaningful co-production for health equity.
**AUTHORS**	Singh, Guddi<br>Salama, Mary<br>Zhu, Hannah

VERSION 1 – REVIEW

**REVIEWER NAME**	Guevara Ramírez, Juan
**REVIEWER AFFILIATION**	University of La Sabana Faculty of Medicine, Faculty of Medicine
**REVIEWER CONFLICT OF INTEREST**	No Conflict of Interest
**AI DISCLOSURE**	Gemini 2.5 Flash was used just for correcting writing style. The ideas and recommendations are original.
**DATE REVIEW RETURNED**	23-Sep-2025

**GENERAL COMMENTS**	Dear authors,<br><br>Thank you for the opportunity to review this manuscript. This is an exceptionally high-quality and highly relevant piece of work for BMJ Paediatrics Open and the broader field of public health. It addresses a critical gap in the Quality Improvement (QI) and co-production literature: how to move past tokenistic involvement to genuinely address structural inequities in paediatric care. The critical honesty about systemic barriers is the article's most significant contribution.<br><br>My recommendation for a major revision is based on the need to strengthen the qualitative methodological presentation and, most importantly from an epidemiological and public health systems perspective, to elevate the discussion on the structural unsustainability of this work, by reviewing the following points:<br><br>1. Foregrounding structural hypocrisy and sustainability or the systems critique.<br><br>The finding concerning the project's reliance on "extensive unpaid and affective labour" leading to burnout for the core team member is arguably the most powerful and critically important finding of the entire paper. This exposes the "structural hypocrisy" of health systems that advocate for equity and co-production without providing the necessary dedicated infrastructure and protected time. This must be the primary message. Then, the abstract and key messages must be slightly reformulated to feature this critical conclusion: meaningful co-production, as implemented, is currently unsustainable for clinical and community leaders without dedicated structural investment.<br><br>Also, the section on "Systemic Barriers and Institutional Constraints"

	must be expanded to deeply unpack the implications of the burnout finding. What funding models and performance metrics need to change for this "relational work" to be recognised, resourced, and protected from leading to burnout? This will give the manuscript a much stronger policy and systems-level impact relevant to the BMJ.<br><br>2. Enhancing qualitative methodological rigor.<br><br>While the theoretical framework is sound, the details of the thematic analysis (or so called RTA) are currently too sparse. Please provide a clearer account of the RTA process: Who was involved in the analysis (e.g. people involved as co-analysts, or only the academic/clinical researchers)? How exactly was the integration/triangulation of data sources (creative artefacts, observational data, textual feedback) handled, and how were they weighted in the final analysis? Provide a brief description of the theme generation process (e.g., was it predominantly inductive, or deductive within the established feminist/ theoretical frame?). This is essential for transparency and reproducibility, as the ones mentioned on the qualitative basic criteria by Lincoln and Guba.<br><br>3. Integrating the process evaluation findings.<br><br>From an epidemiological and QI perspective, the link from process to systemic outcome needs better closure. There is a significant difference in the N of participants (102 YP in Birmingham vs. 14 YP in London). While the authors state the aim was process evaluation, this massive scale difference needs explicit justification, particularly when comparing the suitability and generalisability of the LISTEN UP! Science versus the SHOW UP! Arts models. How does this scale difference impact the conclusions about the models' efficacy? The Logic Diagram (Figure 1) mentions "Changes to local service configuration/provision" and "Evidence of improved trust and relationships... and culture change" as global aims and measures. The discussion must address if was any evidence of tangible systemic change (e.g., a new co-designed protocol, a change in staffing or service delivery model) observed by the time of reporting. If not, this must be highlighted as a limitation of the process evaluation and a strong mandate for future follow-up research.<br><br>4. Terminology consistency (minor revision): Please ensure consistency in key terminology (e.g., "meaningful co-production," "health equity") is maintained throughout the manuscript for clarity.<br><br>5. Figures (minor revision): Ensure that Figure 1 (Logic Diagram) is presented legibly and is clearly referenced in the text, as it is crucial for the reader to understand the authors' theory of change.<br><br>I look forward to reviewing the revised manuscript. If the team addresses these major concerns with the necessary rigor, particularly by foregrounding the critical insights on structural unsustainability and detailing the qualitative methodology, I am confident that this article will be a landmark publication on meaningful co-production for health equity.

**REVIEWER NAME**	Napier-Raman, Sharanya
**REVIEWER AFFILIATION**	The University of Sydney
**REVIEWER CONFLICT OF INTEREST**	No Conflict of Interest

**AI DISCLOSURE**	No, I have not used AI
**DATE REVIEW RETURNED**	03-Oct-2025

**GENERAL COMMENTS**	Thank you for this interesting and well-written paper. Consider having more discussion of what 'marginalised youth' means in this context - how is 'marginalised' defined? Sometimes using this term without addressing the myriad intersecting aspects of disadvantage and strength can be flattening.<br><br>Abstract:<br>- perhaps a clearer opening statement<br><br>Introduction<br>- When you discuss disparities and 'disproportionately poor outcomes' of young people (pg 6, line 7-8), perhaps just say who the comparison is with. Older adult populations? Older adult populations from the dominant culture?<br>- I'm not sure if this will come later, but unpacking what is meant by 'marginalised' in the context of this study would be good<br>- I think you need to explain further the conclusion in lines 11-12 (pg6) that ' Paediatrics thus provides a revealing lens...'<br>- Avoid multiple rhetorical questions<br><br>Methods<br>- The many tables in the methods do make this a bit choppy and in some ways difficult to follow (though I acknowledge this may be the easiest way to present these methods)<br>- How these two 'arms' relate and will be assessed together is a little unclear; why were these two strands used, why was one in Birmingham and the other in London (was there any reasoning behind choosing Listen Up! for Birmingham?).

VERSION 1 – AUTHOR RESPONSE

Reviewer / Editor Comment	Author Response	Location in Revised Manuscript
Overall request to strengthen methodological transparency, systems critique, and sustainability argument.	We thank the Associate Editor and both reviewers for their thoughtful and constructive feedback. We have revised the manuscript throughout to strengthen methodological clarity, deepen the systemic analysis, and foreground sustainability and structural hypocrisy as central findings.	Global revisions throughout manuscript.
Abstract must foreground structural unsustainability, “labour of love vs institutional hypocrisy,” burnout, and policy implications.	The Abstract has been fully revised. It now foregrounds structural hypocrisy, explicitly addresses reliance on unpaid and affective labour, describes burnout among facilitators, and highlights implications for national and organisational policy.	Abstract (all paragraphs).

Reviewer / Editor Comment	Author Response	Location in Revised Manuscript
Key Messages should clearly identify structural hypocrisy and sustainability challenges.	All three subsections have been revised to explicitly reference structural hypocrisy, the emotional/relational labour required, and the need for system-wide investment to sustain equity-focused co-production.	Key Messages section.
Define “marginalised youth”; clarify comparison group; remove rhetorical questions; strengthen conceptual framing.	We clarified the definition of marginalised youth, specified the comparison group in describing disparities, removed rhetorical questions, and strengthened conceptual grounding around epistemic inequity, relational barriers, and the limits of traditional QI.	Introduction → “Health Inequality and the Limits of Improvement Work.”
Explain why paediatrics provides a revealing lens for examining inequity.	We expanded this rationale by describing how paediatrics uniquely encounters lived social determinants, cumulative disadvantage, and the relational nature of inequity.	Introduction (paras. 3–4).
Clarify feminist, relational, and critical underpinnings and how they shaped methodological decisions.	We expanded this section to explicitly articulate how feminist, relational, and community-based theoretical foundations informed all methodological decisions, including data collection, co-production design, and analytic approach.	Methods → Theoretical Orientation.
Clarify rationale for dual-arm design and differences between sites; emphasise real-world opportunistic design.	We added a concise explanation that the pilot intentionally compared two contrasting approaches: LISTEN UP! as a “wide-but-shallow” model (many youth, short encounters) and SHOW UP! as a “deep-but-narrow” model (fewer youth, intensive engagement). We now state clearly that the study was an opportunistic, practice-embedded inquiry operating within real-world system constraints.	Methods → Study Design (paras. 2–4, plus new comparative sentence).
Justify site selection and describe population characteristics.	Expanded to clarify why Birmingham and London were chosen as contexts of pronounced inequity and how school-based recruitment enabled the inclusion of youth least likely to be reached through healthcare settings.	Methods → Settings & Participants.
Provide more detail on data triangulation and multimodal dataset; describe analytic reflexivity; improve flow around tables.	Data Collection is substantially expanded to explain the purpose and function of each data type, the triangulation strategy, and how reflexivity was embedded (journaling, team debriefs, participant validation). Narrative flow and table referencing were improved.	Methods → Data Collection and Tables 1–4.

Reviewer / Editor Comment	Author Response	Location in Revised Manuscript
Provide greater detail on RTA; specify who analysed data; describe analytic phases; clarify inductive/deductive balance; reference Lincoln & Guba.	The Data Analysis section has been fully rewritten. It now describes the six phases of RTA; identifies the analytic team (clinicians, youth partners, artists, social scientists); clarifies triangulation across data types; articulates inductive grounding with deductive theoretical influence; and incorporates Lincoln & Guba's trustworthiness criteria.	Methods → Data Analysis.
Strengthen ethical and reflexive considerations, including relational ethics, emotional labour, and avoidance of tokenism.	The Ethical and Reflexive Considerations section now details relational ethics, layered consent, symbolic and emotional labour, risks of tokenism, and a positionality statement.	Methods → Ethical & Reflexive Considerations.
Clarify differences between LISTEN UP! and SHOW UP! and how site differences shaped findings.	We added explicit statements describing LISTEN UP! as a wide-but-shallow model and SHOW UP! as a deep-but-narrow model, and clarified how these structural differences influenced engagement, mechanisms of change, and emergent barriers.	Results → Material Matters.
Ensure clarity of 3 E's, 3 I's, impacts, and relational values (3 Rs, 3 REALs).	These sections have been refined to ensure conceptual clarity, smoother narrative linking, tighter definitions, and consistent referencing of tables.	Results → 3 E's, 3 I's, Impacts, Values.
Strengthen systems critique; highlight sustainability and burnout; link findings to governance, resource allocation, and policy.	The Discussion now more fully analyses structural hypocrisy, the burden of unpaid relational labour, workforce pressures, governance gaps, and implications for systemic investment and national policy.	Discussion → Systemic Barriers; Transformative Praxis; Limitations.
Remove rhetorical questions and ensure claims are well supported.	Rhetorical questions have been removed or converted into clear statements; conceptual claims are now more explicitly grounded in literature and findings.	Discussion (global edits).
Ensure the Conclusion reflects structural critique, sustainability challenges, and implications for practice and policy.	The Conclusion now emphasises structural hypocrisy, the need for national resource commitments, and frames co-production as moral infrastructure rather than an optional intervention.	Conclusion (fully revised).
Improve clarity, legibility, and referencing of tables and diagrams.	All figures and tables are checked for clarity, consistent labelling, and correct referencing.	Manuscript tables & figures updated.

Reviewer / Editor Comment	Author Response	Location in Revised Manuscript
Ensure Logic / Driver Diagram is clearly referenced and legible.	The diagram has been revised for clarity and is now explicitly referenced in the Methods.	Updated diagram file.

VERSION 2 – REVIEW

**REVIEWER NAME**	Guevara Ramírez, Juan
**REVIEWER AFFILIATION**	University of La Sabana Faculty of Medicine, Faculty of Medicine
**REVIEWER CONFLICT OF INTEREST**	No Conflict of Interest
**AI DISCLOSURE**	ChatGPT 5.3 was used to assist with language editing, structure, and clarity of the peer review.
**DATE REVIEW RETURNED**	14-Mar-2026

**GENERAL COMMENTS**	Thank you for the opportunity to review this manuscript, which topic is important and aligned with current efforts to improve equity and patient engagement in healthcare systems. However, in its current form the manuscript requires substantial revision before it can be considered for publication.<br><br>1. Although the study is described as a qualitative process evaluation using reflexive thematic analysis, the manuscript lacks sufficient detail to allow readers to fully understand how the analysis was conducted. For example:<br><br>- The number of interviews conducted is not clearly reported.<br>- It is unclear how many participants contributed to surveys, video diaries, or reflective materials.<br>- The analytical process remains largely conceptual rather than procedural.<br>- Greater transparency is needed regarding the volume and type of data analysed, how codes were generated, how disagreements between coders were handled, how themes were finalised.<br><br>2. Several sections of the findings (e.g., the “3 Es”, “3 Is”, and “3 Rs” frameworks) appear highly structured in ways that suggest post-hoc conceptual framing rather than inductively derived themes. The manuscript should clarify whether these categories emerged directly from the data, or whether they were constructed analytically by the research team.<br><br>More direct linkage between quotes, codes, and themes would strengthen the empirical grounding of the results. A central aim of the study appears to be comparison between two approaches (LISTEN UP and SHOW UP). However, the results sections often blend findings across sites rather than presenting a clear comparative analysis. The authors should consider clarifying:<br><br>- which findings were specific to each model<br>- which findings were shared across both models<br>- whether one model appeared more feasible or impactful.

	3. At times the manuscript adopts language that reads as advocacy rather than scientific analysis. For example, phrases such as “institutional hypocrisy”, “creative discomfort”, “labour of love”. It may reduce the perceived analytical neutrality of the work.<br><br>4. The discussion would benefit from a slightly more balanced tone that distinguishes clearly between empirical findings and normative interpretation. While the authors acknowledge the small scale of the pilot, the manuscript would benefit from deeper reflection on how transferable these findings may be beyond the UK NHS context, what conditions would be required for replication, and whether the approach is feasible in resource-limited settings.<br><br>5. Some tables repeat information already described in the text and could potentially be condensed.<br><br>6. The introduction could be shortened slightly to improve focus.<br><br>7. Some quotations appear long relative to the analytic commentary provided. Consider balancing quotations with deeper interpretive analysis.<br><br>8. The manuscript occasionally repeats conceptual frameworks (e.g., the “3 Es” and “3 Rs”) across multiple sections.<br><br>9. A clearer explanation of the distinction between quality improvement activity and research would help readers unfamiliar with UK governance structures.

**REVIEWER NAME**	Napier-Raman, Sharanya
**REVIEWER AFFILIATION**	The University of Sydney
**REVIEWER CONFLICT OF INTEREST**	No Conflict of Interest
**AI DISCLOSURE**	No, I have not used AI
**DATE REVIEW RETURNED**	17-Mar-2026

**GENERAL COMMENTS**	Thank you for the thorough revisions and responses. I think this paper needs to be cut down and edited - word count is very long, and there is a reliance on tables to present results which makes it very choppy.<br><br>I would recommend adding a citation after the definition of marginalised youth (Introduction).<br><br>I would recommend getting rid of the amount of results written as dot points and in tables. I understand this paper is very long - perhaps efforts could be made to cut down somewhere, but I feel that the results should be written primarily in paragraph form. For a qualitative paper, there are a lot of tables. Would recommend trying to streamline this piece by converting some of these tables to paragraphs. (This may mean you have to cut words, which I think you could from the methods section and perhaps discussion).<br><br>Please also ensure that there is consistency with participant details provided for each quote -- Tables 5 and 6 indicate who quotes are from (e.g. 'junior doctor'), but others do not. This is important information

	While the Discussion section is good and thorough, it is very long. I would try to make the paper as a whole more concise. Part of the issue with this is the number of tables does break up the writing significantly and make this less easy to read.<br><br>Formatting issues -- a few different fonts, and sometimes text colour and size variations.<br><br>If you can make some of the above minor revisions, that would be great.

VERSION 2 – AUTHOR RESPONSE

Reviewer Comment	Response	Location of Change
**Reviewer 1: Greater transparency needed regarding volume and type of data analysed. Number of interviews, surveys, video diaries and reflective materials not clearly reported.**	Thank you for this suggestion. We have substantially expanded methodological transparency by adding a detailed data corpus table describing all data sources, approximate volume of material, participant involvement and analytic purpose. This provides readers with a clearer understanding of the breadth and nature of the dataset used in the process evaluation.	Methods – Data Collection; revised Data Corpus table.
**Reviewer 1: Greater transparency required regarding coding, theme development and analytic process.**	We have revised the Data Analysis section to provide a fuller description of the Reflexive Thematic Analysis process, including familiarisation, coding, theme development, triangulation, reflexive synthesis and collaborative interpretation. We have also clarified how diverse disciplinary perspectives within the research team informed analysis.	Methods – Data Analysis.
**Reviewer 1: Clarify triangulation across creative outputs, observations and textual materials.**	Additional detail has been added describing how multiple data sources were intentionally combined to capture different dimensions of participation, meaning-making and system interaction. We have clarified the role of triangulation across creative, observational and textual data sources.	Methods – Data Collection and Data Analysis.
**Reviewer 1: Clarify how disagreements between coders were handled.**	We have clarified that analysis followed a reflexive thematic analysis approach, in which differences in interpretation were explored through discussion and reflexive engagement rather than treated as problems requiring consensus or inter-rater reliability.	Methods – Data Analysis.

Reviewer Comment	Response	Location of Change
**Reviewer 1: Reference Lincoln and Guba criteria.**	We have added an explicit account of credibility, dependability, confirmability and transferability, and explained how these principles informed the study.	Methods – Data Analysis.
**Reviewer 1: Clarify whether the 3 Es, 3 Is and 3 Rs emerged directly from data or were post-hoc conceptual constructions.**	We have explicitly clarified that these frameworks were not pre-existing coding categories nor discrete participant-generated themes. Rather, they were developed during later-stage reflexive synthesis as interpretive heuristics to organise recurrent patterns identified across multiple data sources and sites.	Methods – Data Analysis.
**Reviewer 1: More direct linkage needed between quotes, themes and findings.**	Findings tables have been revised and streamlined to provide clearer alignment between themes, interpretation and illustrative participant quotations. The narrative accompanying each section has also been revised to strengthen the connection between empirical data and interpretation.	Results – all findings sections and revised tables.
**Reviewer 1: Comparative analysis between LISTEN UP! and SHOW UP! requires clarification.**	We have strengthened comparative reporting throughout the manuscript. The study design now explicitly explains the rationale for comparing two intentionally contrasting co-production models, and the findings now distinguish shared mechanisms from site-specific strengths and constraints. We also discuss trade-offs between breadth and depth of engagement in the Discussion.	Study Design; Findings introduction; Discussion – Transferability and implications.
**Reviewer 1: Language occasionally reads as advocacy rather than analysis (e.g. institutional hypocrisy, labour of love).**	We carefully reviewed the manuscript and refined several terms to ensure analytical clarity. Where the concept of structural hypocrisy is retained, we now define it explicitly and situate it within relevant theoretical literature concerning implementation gaps, institutional non-performativity and invisible caring labour. This strengthens the conceptual justification for the interpretation.	Discussion – Hidden Cost of Meaningful Co-production; Transferability and Implications.
**Reviewer 1: Discussion should more clearly distinguish empirical findings from normative interpretation.**	The Discussion has been substantially reorganised. Findings are now interpreted sequentially through four sections addressing epistemic repair, barriers and sustainability, systemic belonging, and implications for practice and policy. This	Entire Discussion section.

Reviewer Comment	Response	Location of Change
	creates a clearer distinction between empirical observations and broader interpretation.	
**Reviewer 1: Discuss transferability, feasibility and replication beyond the NHS context.**	We expanded discussion of transferability, feasibility, breadth-versus-depth trade-offs, and adaptation to different contexts, including resource-constrained settings.	Discussion – Transferability, Limitations and Implications.
**Reviewer 1: Some tables repeat information already presented in text.**	We undertook a major review of all tables. Redundant tables were removed or consolidated, and remaining tables were revised to provide distinct analytic value rather than duplicate narrative content.	Tables throughout manuscript.
**Reviewer 1: Introduction could be shortened.**	The Introduction has been substantially streamlined to improve focus while retaining the theoretical and empirical rationale for the study.	Introduction.
**Reviewer 1: Some quotations are long relative to analytic commentary.**	Quotations have been reviewed and selectively shortened. Additional analytic interpretation has been added where appropriate to strengthen the connection between data and findings.	Results tables and findings narrative.
**Reviewer 1: Conceptual frameworks repeated across multiple sections.**	We reduced repetition and simplified the manuscript structure. Several frameworks were consolidated and integrated into existing findings rather than repeatedly reintroduced across sections.	Results and Discussion.
**Reviewer 1: Clarify distinction between quality improvement and research.**	We expanded the Study Design and Ethical Considerations sections to explain the quality improvement nature of the project, its governance arrangements and the rationale for the methodological approach adopted.	Study Design; Ethical and Reflexive Considerations.
**Reviewer 2: Manuscript is overly long and fragmented by excessive tables.**	We undertook substantial restructuring, reducing duplication, consolidating tables, simplifying presentation and streamlining narrative sections. The manuscript is now substantially shorter and more cohesive.	Throughout manuscript.
**Reviewer 2: Add citation after definition of marginalised youth.**	Appropriate citation added.	Introduction.

Reviewer Comment	Response	Location of Change
**Reviewer 2: Convert some table content into prose and improve flow.**	Several tables were removed, merged or consolidated, and findings have been more fully integrated into the narrative while retaining key evidence and quotations.	Results and Tables.
**Reviewer 2: Standardise participant attribution for quotations.**	Participant descriptors have been standardised throughout all tables and findings sections.	Results tables.
**Reviewer 2: Discussion is overly long.**	The Discussion has been substantially rewritten and reorganised to reduce repetition, improve flow and sharpen the paper's central argument.	Discussion.
**Reviewer 2: Formatting inconsistencies.**	Formatting, font and style inconsistencies have been reviewed and corrected throughout the manuscript.	Entire manuscript.

VERSION 3 – REVIEW

**REVIEWER NAME**	Guevara Ramírez, Juan
**REVIEWER AFFILIATION**	University of La Sabana Faculty of Medicine, Faculty of Medicine
**REVIEWER CONFLICT OF INTEREST**	No Conflict of Interest
**AI DISCLOSURE**	ChatGPT (OpenAI) was used to assist in improving the clarity, grammar and structure of the reviewer comments. All scientific assessment, critical appraisal, and final review content were independently performed and verified by the reviewer.
**DATE REVIEW RETURNED**	22-Jun-2026

**GENERAL COMMENTS**	Consider including a brief demographic table summarising participant characteristics.<br><br>Future research directions could be expanded, particularly regarding long-term impacts on young people, clinicians, and organisational change.
